# A Phytobezoar Causing Terminal Ileal Obstruction Following Revision Bariatric Surgery: A Case Report

**DOI:** 10.7759/cureus.37353

**Published:** 2023-04-10

**Authors:** Siddharth Sankar Das, Zaid AbdelAziz, Walid Zakaria Abdelhamid M Bondok, Farah Ibrahim B Juma, Feras Hamid A Khatib

**Affiliations:** 1 General Surgery, Dubai Hospital, Dubai, ARE

**Keywords:** bariatric surgery, enterotomy, intestinal obstruction, phytobezoar, rygb

## Abstract

Bezoars are a rare complication causing small bowel obstruction. A phytobezoar causing terminal ileum obstruction following a Roux-en-Y gastric bypass (RYGB) is extremely rare. A middle-aged woman with post-sleeve gastrectomy weight regain, converted to RYGB, presented 17 months after surgery with obstructive symptoms due to an impacted phytobezoar in the terminal ileum. Diagnostic laparoscopy, enterotomy, and extraction of the large impacted phytobezoar from the terminal ileum relieved the obstruction. Swallowing improperly masticated food in altered gastrointestinal anatomy due to RYGB can cause a phytobezoar in any part of the gastrointestinal tract. These patients need proper nutritional counseling and psychological evaluation to prevent this rare complication.

## Introduction

Phytobezoars, though rare, require prompt intervention when they cause small intestine obstruction. Bezoar formation after gastric bypass is extremely rare. Accelerated gastric pouch emptying insufficiently masticated food in altered gastrointestinal anatomy due to Roux-en-Y gastric bypass (RYGB) can cause a phytobezoar in any part of the gastrointestinal tract [1]. Bezoars presenting with intestinal obstruction following gastric bypass require prompt diagnosis and intervention to relieve the obstruction. A laparoscopic approach can relieve obstruction and facilitate early postoperative recovery.

## Case presentation

The patient was a 50-year-old Emirati woman with an initial weight of 132 kg and body mass index (BMI) of 46.80 kg/m^2^ with comorbidities of hypertension, dyslipidemia, and back pain. She underwent laparoscopic sleeve gastrectomy in December 2014. She reduced her weight to 75 kg following a sleeve gastrectomy, then increased her weight to 98 kg and her BMI to 36.17 kg/m^2^, and developed reflux symptoms. Hence, she underwent RYGB in August 2017. During the RYGB, 30 ccs of gastric pouch were created, and 80 cm of biliopancreatic limb and 130 cm of roux limb were constructed. A gastrojejunostomy was created with a circular stapler, and a jejunojejunostomy (j-j anastomosis) was created with a linear stapler. Both the j-j anastomosis mesenteric defect and Peterson’s defect were closed. After the RYGB, she reduced her weight to 80 kg and BMI to 28.36 kg/m^2^, and her reflux symptoms resolved immediately after revision surgery. Postoperatively, she attended the bariatric clinic without any complaints.

**Figure 1:**
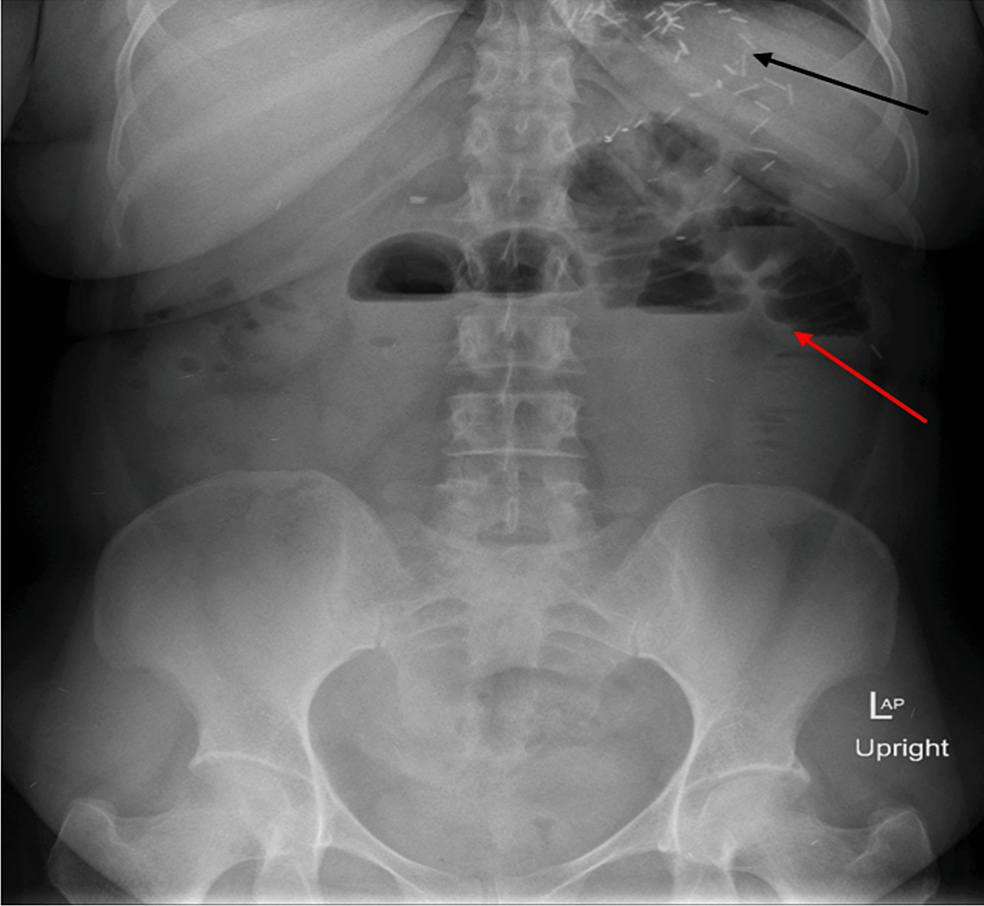
X-ray of the abdomen showing multiple air-fluid levels (red arrow) and staples of gastric bypass (black arrow).

On January 14, 2019, she presented to the emergency department with a complaint of acute onset of epigastric pain associated with multiple episodes of vomiting for the last two days. She had never experienced this type of pain before. Initially, she went to another clinic and was given analgesics and an antiemetic and referred to our emergency department. She was afebrile and tachycardic but maintained her blood pressure. On examination, her abdomen was mildly distended, with tenderness mainly in the epigastric and umbilical regions, without signs of peritonitis. The bowel sounds were slightly exaggerated, and she had been passing gas per anus but no stool for one day. A laboratory test showed leukocytosis (12.2 × 10^3^/µL) with absolute neutrophilia (8.3 × 10^3^/µL). An X-ray of the abdomen showed small dilated bowel loops with multiple air-fluid levels (Figure 1).

**Figure 2:**
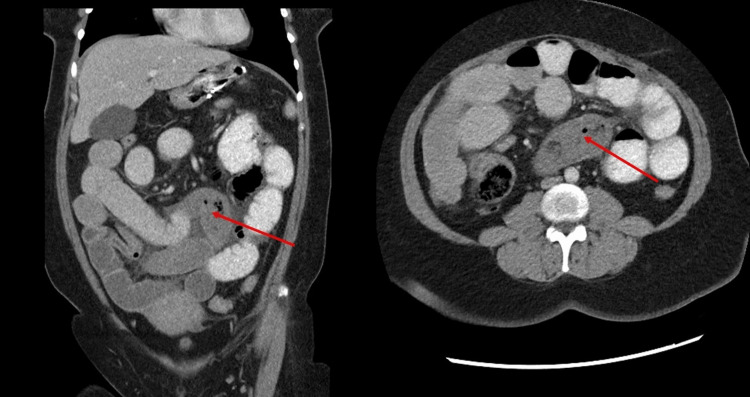
A CT scan of the patient’s abdomen showing an obstruction in the small intestine (red arrow).

She vomited again in the emergency department; the vomit contained small intestine contents. A nasogastric tube was inserted, which brought out around 300 mL of bilious content. A computed tomography (CT) scan with contrast was performed, which showed dilated jejunal and proximal ileal loops with multiple air-fluid levels without passage of oral contrast to the distal ileum. A string of beads sign and a small bowel feces sign in the distal ileum suggested obstruction (Figure 2).

**Figure 3:**
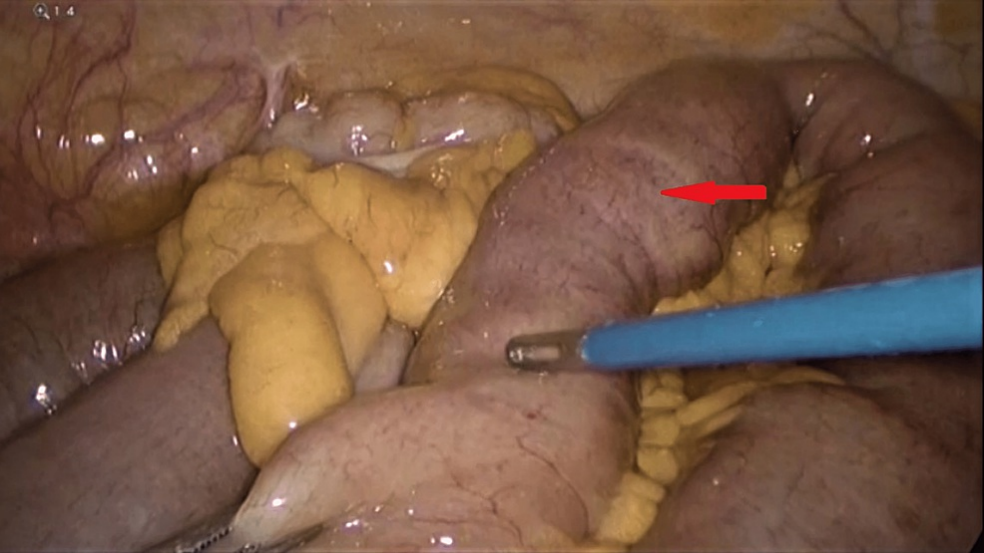
Identifying the impacted bezoar in the terminal ileum (red arrow).

**Figure 4:**
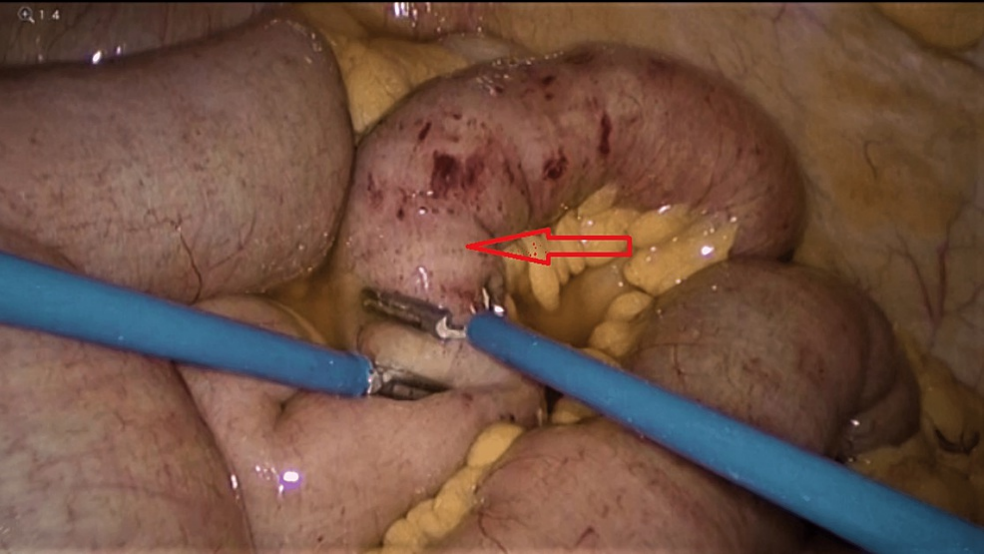
Trial milking of the bezoar from the terminal ileum to the cecum (red arrow).

**Figure 5:**
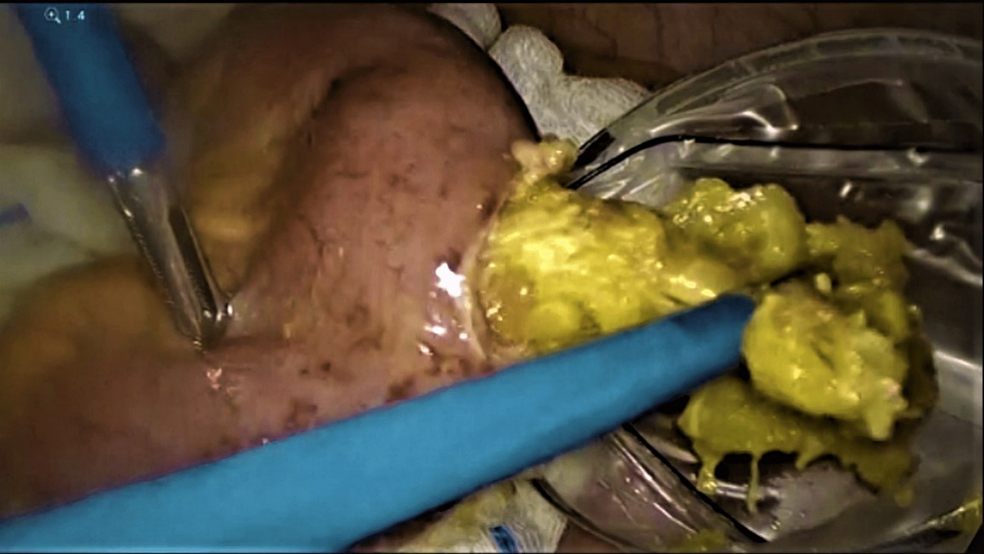
Laparoscopic extraction of the phytobezoar into the Endobag.

The CT scan findings were discussed with the patient, and an emergency diagnostic laparoscopy was planned. During the diagnostic laparoscopy, the gastric pouch was found to be of normal size, but there was distention of jejunal loops to the distal ileum. The j-j anastomosis was dilated but patent. In the distal ileum, there was a transitional zone around 40 cm from the ileocecal junction with an intraluminal globular mass obstructing the distal ileum (Figure 3). With a bowel grasper, the intraluminal mass was felt; it occupied around 10 cm of the distal ileum with a collapsed distal part. The entire large bowel was found normal in caliber without any visible pathology. We attempted but failed to milk the distal ileum mass to propel it through the ileocecal valve up to the cecum so the patient could expel it subsequently (Figure 4). We failed to dis-impact the intraluminal mass from the distal segment of the ileum with repeated trials. Hence, the dilated ileum proximal to the obstruction was clamped with a bowel grasper, and an enterotomy was performed over the obstruction, opening the ileum longitudinally. An intraluminal bezoar was found and extracted carefully with the help of a gasper and a Babcock, preventing spillage. The food bolus mixed with stool was collected in an Endobag to prevent contamination (Figure 5). After the removal of the complete bezoar mass, any additional extension was looked for but not found. The enterotomy was closed with a 3-0 PDS continuous suture. A thorough abdominal lavage was performed before completing the procedure. On examination, the bezoar was found to contain multiple orange segments mixed with food particles and stool.

The patient’s postoperative recovery was smooth. She started tolerating liquids from the first postoperative day, progressed to a soft diet on postoperative day two, and was discharged from the hospital on the third postoperative day. When we spoke to her during the postoperative period, she admitted to consuming orange segments directly without proper chewing three days before while in the office during her busy working hours. She was advised to chew her food properly before swallowing to prevent such a complication in the future. She was also advised to regularly follow up with the bariatric and dietician clinics.

## Discussion

Post-gastric bypass patients need specific diet plans in the preoperative and early and late postoperative phases. They should strictly abide by the diet plan and visit a dietician for any change or modification according to their weight loss pattern in the postoperative phase. The post-RYGB weight loss pattern and any development of complications depend on the patient’s discipline and consistency in her diet quality and quantity.

Immediately after surgery, post-gastric bypass patients should follow their dietician’s plans and should be limited to liquid diets only, then progressing to a puree diet. After one month, they should start a soft diet, with proper mastication. After surgery, the newly formed gastric pouch restricts intake, and the patient should be careful while eating and drinking. Once they understand and get familiar with the eating pattern, it becomes easier for them to swallow food of different textures. Usually, after six months to one year, they can eat every type of food. Hence, this complication is prone to occur after six months of surgery if the patient swallows food without proper mastication.

Bezoars are retained undigested bolus materials in the gastrointestinal tract. A bezoar predominantly consists of food (phytobezoar) or hair (trichobezoar). Factors contributing to the development of phytobezoars include high-fiber food rich in cellulose, less gastric acid from the small stomach pouch after RYGB, improper mastication, and delayed gastric emptying post-RYGB [1]. In our case, improper mastication was the main factor in the formation of phytobezoar, as she swallowed orange segments without chewing properly. Proper education about the importance of chewing each bite of a meal after RYGB can prevent phytobezoar formation. All our patients are counseled preoperatively about their new dietary adaptation during the postoperative period. All our patients undergo psychological analysis during the pre- and postoperative periods. This patient underwent the same dietary counseling and psychological evaluation preoperatively, which was reinforced after the second surgery.

Internal hernia is the most common cause of small intestinal obstruction following RYGB [2-5], followed by interloop adhesion, stenosis, or a kink at the j-j anastomosis, roux limb stricture, volvulus, incarcerated ventral hernia, and intraluminal hematoma [6,7]. Post-RYGB small bowel obstruction due to a phytobezoar is rare (<1%). Post-RYGB small bowel obstruction is diagnosed mainly by presentation symptoms, clinical examination, laboratory examinations, and supportive radiological studies, including a CT scan of the abdomen. If the obstruction affects the alimentary limb, the usual presentation includes reflux symptoms and significant hyperemesis. Obstruction of the biliopancreatic limb usually presents with tachycardia, elevated liver enzymes, and hyperamylasemia. Presentation with bilious vomiting suggests common limb obstruction or, rarely, obstruction due to a gastrogastric fistula [3]. Obstruction due to a bezoar usually occurs more than six months later in post-RYGB patients and can present with abdominal pain, nausea, vomiting, ulceration, and a bleeding gastrointestinal tract. A small bowel phytobezoar can be identified using a CT scan, whereas upper gastrointestinal fluoroscopy or endoscopy can show bezoar formation in the gastric pouch. With high clinical suspicion of small bowel obstruction with negative radiological results, surgical exploration is still recommended [3,8].

Treatment options for phytobezoars include chemical dissolution, upper gastrointestinal endoscopic fragmentation and extraction, or surgical intervention. Patients with acute obstruction with an impacted bezoar in the distal small bowel usually need laparoscopic intervention or, if failed, conversion to open exploration. For a phytobezoar in the distal ileum, an initial attempt of intraluminal fragmentation by external compression, milking through the ileocecal valve, to relieve the obstruction should be made to prevent enterotomy [9]. If this maneuver fails, an enterotomy and bezoar extraction should be performed.

Post-gastric bypass patients need regular follow-ups with a nutritionist regarding their diet plan and their dietary habits. On enquiring in the postoperative period, this patient accepted consuming orange segments without proper mastication, three days before developing obstructive symptoms, which emphasizes the need for regular follow-ups with a dietician to discuss their eating habits and any modification of diet plans according to their job demand. Patients also need regular follow-ups with a psychologist for assessment and counseling to adjust to their changed lifestyle and environment.

Although phytobezoars are a rare complication, they should be considered a differential diagnosis in post-gastric bypass patients presenting with obstructive symptoms. Going through the literature, this is the first reported case of a phytobezoar causing small bowel obstruction following revision bariatric surgery. For any suspicion of obstruction, diagnostic laparoscopy should be maintained as a prompt intervention option to rule out any other extrinsic cause of obstruction. Radiologically imaging and diagnosing phytobezoars is key to early intervention [10,11].

## Conclusions

Phytobezoars are a rare cause of small bowel obstruction following revision gastric bypass. They should always be suspected in patients presenting with a small bowel obstruction following RYGB due to their anatomical alteration and change in dietary habits. Laparoscopic management of bezoar-induced intestinal obstruction by experienced surgeons is feasible with early recovery. These patients need regular nutritional counseling and psychiatric evaluation to prevent further bezoar formation.
